# The conidial mucilage, natural film coatings, is involved in environmental adaptability and pathogenicity of *Hirsutella satumaensis* Aoki

**DOI:** 10.1038/s41598-017-01368-1

**Published:** 2017-05-02

**Authors:** Jiaojiao Qu, Xiao Zou, Jianping Yu, Yeming Zhou

**Affiliations:** 1 Key Laboratory of Green Pesticide and Agricultural Bioengineering, Ministry of Education, Guizhou University, Guiyang, 550025 China; 2 Institute of Fungus Resources, College of Life Sciences, Guizhou University, Guiyang, 550025 China; 3 College of Life Sciences, Guizhou University, Guiyang, 550025 China; 4 Institute of Entomology, Guizhou University, Guiyang, 550025 China

## Abstract

The *Hirsutella* genus is very special asexually-reproducing pathogens of insects by reduced sporulation, host specificity and spores covered by a thick mucilage layer. However, the ecological function of conidial mucilage remains elusive. In this study, the possible ecological role of conidial mucilage from the entomopathogenic fungus *Hirsutella satumaensis* was functionally investigated through tolerance, adherence and insect bioassays involving aerial conidia (AC) and mucilage-free conidia (MFC). Measurements of hydrophobicity using microbial adhesion to hydrocarbons (MATH) indicated that mucilage is main contributor to the surface hydrophobicity of AC. When subjected in tolerance assays to extreme temperatures, high chemical pressure, extended exposure to ultraviolet radiation and cold stress, AC produced more colonies, exhibited higher conidiation and germination percentages than those of MFC. In adhesion assays, MFC displayed an approximately 40% reduction in adherence to locust, dragonfly cuticle and onion epidermis when washed with 0.05% Tween 20. Similarly, *Galleria mellonella* and *Plutella xylostella* larvae infected with mucilage-producing AC experienced a relatively higher mortality rate. Our findings suggest that mucilage is critical to the ecological adaptability of *H. satumaensis*, where it plays positive roles on maintenance of spore surface hydrophobicity, enhancement of spore resistance to extreme environments and strengthening of spore adhesion and host pathogenicity.

## Introduction

Extracellular mucilaginous material (ECMM) is secreted by a wide range of organisms, including fungi, bacteria and algae^1–3^. At present, the proposed roles of ECMM include attachment of spores or mycelium to various substrates and protection^4^. Boucias *et al*.^5^ have reported that the spores of terrestrial entomopathogenic fungi, such as *Verticillium lecanii*, as well as those of certain species of the family Entomophthorales, are covered by an amorphous mucilage that facilitates adhesion of the spore to the host cuticle. Mondal and Parbery^6^ confirmed that the mucilaginous spore matrix protects spores of *Colletotrichum* species against temperature extremes. Other roles of ECMM may include the storage of nutrients used by the fungus during starvation^7^.

One type of ECMM, the mucilage of spores, is ubiquitous in *Hirsutella* Pat., a globally distributed genus of pathogens of arthropods, mites and nematodes as well as the anamorphs of the fungus *Ophiocordyceps* Sung., a popular traditional medicine and nutritious food in many Asian countries^8–10^. *Hirsutella* species are distinguished from other asexually typified genera with wide host ranges by the presence of basally swollen or subulate phialides that taper to an apex where a mucilaginous packet of one or several conidia forms^11^. This mucilage may play an important role in fungal access to nutrients. For example, mucilage helps *H. rhossiliensis* more effectively adhere to the surface of passing nematodes. After penetration of the cuticle directly beneath the adhering spore, a bulbous infection hypha forms from which secondary hyphae develop. Interestingly, mycelia of nematophagous fungi tend to grow and attract nematodes in the environment, with adhesion occurring thereafter^12^. Jansson *et al*.^13^ studied the process by which the end-parasitic fungus *Drechmeria coniospora* uses mucilage spores to attract host nematodes. They found that the quantity of spore mucilage is positively correlated with attraction and adhesion to nematodes. The adhesive material can also be observed through a microscope. Using electron microscopy, Saikawa *et al*.^14^ examined exterior adhesive on the spores of the end-parasitic fungus *Harposporium oxycoracum* and concluded that this substance is made up of a polysaccharide-protein complex.

Numerous studies have attempted to resolve and characterize the compounds in mucilage of plant pathogenic fungi to facilitate investigation of their function^15, 16^. The major component of the mucilaginous matrix produced by the fungus *Colletotrichum graminicola* is a group of high-molecular-weight glycoproteins composed of oxygen-linked oligomers of rhamnose and mannose and high levels of hydrophobic and hydroxylic amino acids^17^. Preliminary tests have indicated that a species of *Hirsutella* parasitic on spruce budworm larvae produces a highly viscous metabolite, possibly a polysaccharide, when grown in liquid culture. Polysaccharides produced by some parasitic fungi have since been reported to be able to cause significant reactions in the host^18^.

At present, there are no reports on ecological role of conidial mucilage from the insect pathogen *Cordyceps*-related genus comprehensively. Previous work mainly focus on the fungal cell surface material, which have important roles in processes such as morphological development and pathogenicity, identification for host adhesion and immune evasion^19, 20^. While related genes such as *M. anisopliae MAD1* ^21^, *Candida* adhesins *Int1* ^22^ and *Als1p* ^23^ and *S. cerevisiae Flo11* ^24^ have been reported to be involved in both adhesion and morphogenesis, research on the structure, function and regulatory mechanism of fungi appendages of *Ophiocordyceps* is limited. Whether in the asexual generation (*Hirsutella*) or the sexual stage (*Cordyceps*), mucilage appears during the spore maturation process. Establishing surface contact between mucilage and host (e.g., arthropods or plant roots) is a crucial step for host infection. Elucidation of the ecological role of mucilage is thus needed. In this study, we discerned the ecological functions and significance of conidial mucilage of *H. satumaensis* by studying the effects of mucilage on conidial growth under unsuitable environmental conditions, adhesion rate in different media and virulence to nymphs of *Galleria mellonella* and *Plutella xylostella*.

## Results

### Characterization of mucilage and hydrophobic effect

**Figure 1:**
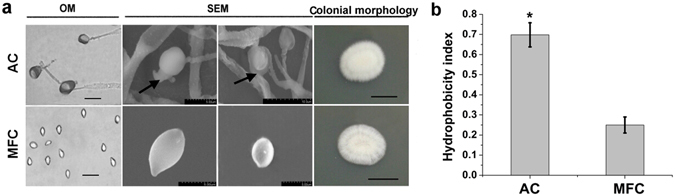
Morphological observing and hydrophobic testing of two spores from *Hirsutella satumaensis*. (**a**) Using LM and SEM image of AC and MFC respectively, the mucilage on AC can be clearly observed no matter under LM or SEM, indicating with the black arrows in the figure. The right shows the colonial morphology of two spores on PDA incubated for 14 days. LM bar = 10 μm, SEM scale bars are in micrographs, colony bar = 1 cm. (**b**) Cell surface hydrophobicity of the two spores assessed by MATH assay. AC is clearly hydrophobic (0.698 ± 0.06) and MFC become hydrophilic removing the mucilage (0.25 ± 0.04). Statistical analysis was performed using a one-way ANOVA. Error bars are standard deviations of three trials and panels are representative of at least 2 independent experiments, *p < 0.05.

Before washing, mucilage amorphously distributed on spore surfaces in 2.0–10 μm clumps (black arrow, Fig. 1a) was readily and deeply stained with lactic acid-phenol-cotton blue solution in contrast to spores. After adding 0.1% β-mercaptoethanol as a reducing agent, mucilage was completely isolated from spores with wash buffer, identified on the basis of its regular shape and smooth surface, and less stained (Fig. 1a). Other than the fact that AC were larger than MFC under an OM because of the presence of mucilage in the former, no significant differences in colonial morphology were observed between the two spore types, which suggests that the mucilage has no effect on colony morphogenesis (Fig. 1a). To determine the composition of mucilage, mucilage extracts were assayed for protein by the Coomassie brilliant blue method. Extraction of mucilage with a solution of 0.1% β-mercaptoethanol at 4 °C for 12 h released approximately 0.105 mg protein ml^−1^ into the washing supernatant (see Supplementary Fig. 1). In addition, the Alexa Fluor 488-labeled lectin concanavalin A specific to α-mannopyranosyl and α-glucopyranosyl residues was bound to the mucilage of *H. satumaensis* AC (see Supplementary Fig. 2). These data indicate that the mucus contains substances such as proteins, oligosaccharides and mannose.

The microbial adhesion to hydrocarbons (MATH) assay (Fig. 1b), in which cells are partitioned between two immiscible solutions (water and hexadecane), was used to assess the cell surface hydrophobicity of the two different *H. satumaensis* spore types. In this assay, entities with HI = 0.7 (where HI is the number of cells in the organic phase divided by the total number of cells) are considered to be hydrophobic^25^. AC were clearly hydrophobic and distributed into the organic phase (HI = 0.698 ± 0.06); in contrast, MFC were hydrophilic, being predominantly localized into the aqueous phase (HI = 0.25 ± 0.04). This result first demonstrates that mucilage is the main contributor to the surface hydrophobicity of AC of *H. satumaensis*.

### Heat tolerance test

**Figure 2:**
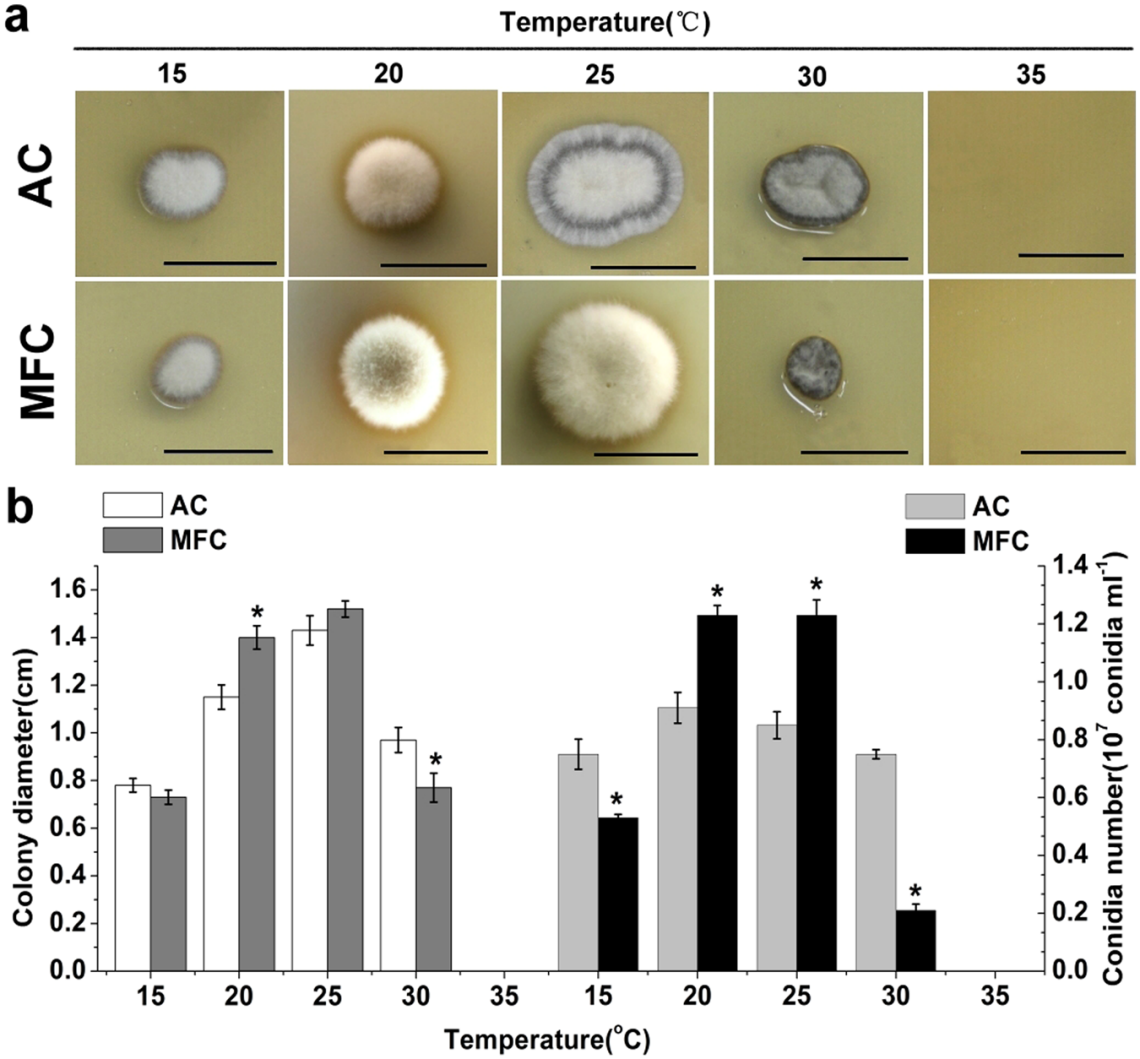
The heat tolerance experiment of two spores from *Hirsutella satumaensis*. (**a**) Colonial morphology of two spores at different temperature ranging from 15 to 30 °C. At 15 °C and 30 °C, colony diameter of MFC is smaller than AC, but which is no difference at 20 °C and 25 °C. Bar = 1 cm. (**b**) Statistics of two spores colony diameter and conidiation at different temperature. Analogously, the results of conidiation were consistent with the colony diameter. Statistical analysis was performed using a one-way ANOVA. Error bars are standard deviations of three trials and panels are representative of at least 2 independent experiments, *p < 0.05.

In this study, temperatures suitable for *H. satumaensis* spore growth ranged from 15 to 30 °C, with optimum values of 20 to 25 °C (Fig. 2). When grown at 15 °C and especially 30 °C, pigment accumulation was evident and dark brown mycelium extended from the colonial margins into the medium (Fig. 2a). Colony diameter and conidiation per 1.33 cm^2^ were used as indices to evaluate mucilage influence on the temperature stress resistance of spores (Fig. 2b). At 30 °C, MFC produced approximately 70% fewer conidia (0.21 ± 0.022 × 10^7^ conidia ml^−1^) than AC (0.75 ± 0.016 × 10^7^ conidia ml^−1^); similar results were observed at 15 °C. An opposite effect was observed within the optimum temperature range of 20 to 25 °C: conidiation of AC (0.85 ± 0.047 × 10^7^ conidia ml^−1^) was less than that of MFC (1.23 ± 0.053 × 10^7^ conidia ml^−1^). Although the colonial morphology of the two types of *H. satumaensis* spores formed on PDA medium was amorphous, the effect of temperature on colony diameters was similar to that observed for conidiation.

### NaCl tolerance test

**Figure 3:**
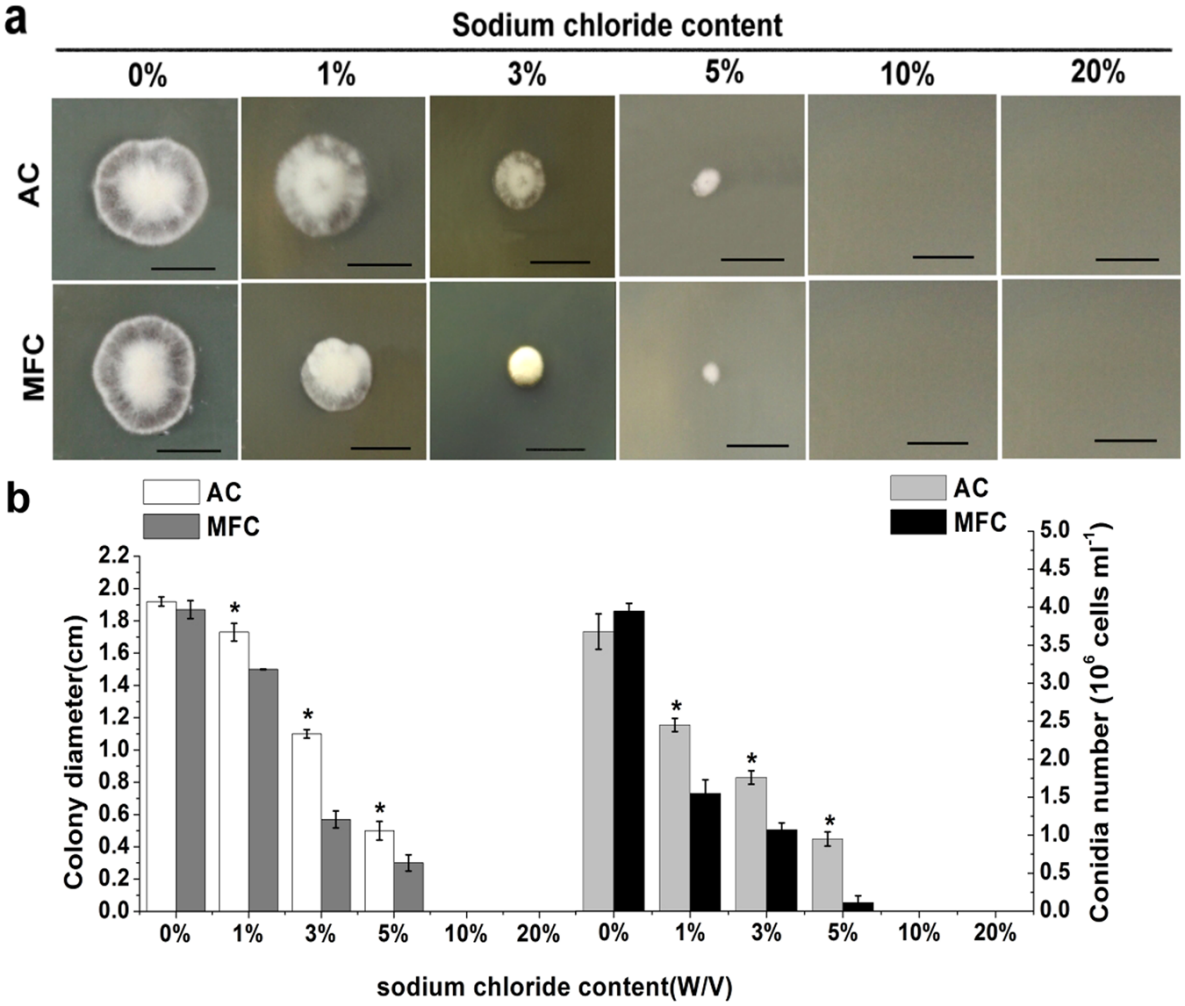
Sodium chloride tolerance test of two spores from *Hirsutella satumaensis*. (**a**) Colonial morphology of two spores at increasing NaCl content. MFC is more susceptible to NaCl and experience drastically depressed growth, with no colonies evident at a NaCl concentration of 10% (w/v). Bar = 1 cm. (**b**) Statistics of two spores colony diameter and conidiation on NaCl medium rangeing from 0% to 10%. The MFC display an approximately 80% reduction in conidiation relative to AC, particularly at 5%. Statistical analysis was performed using a one-way ANOVA. Error bars are standard deviations of three trials and panels are representative of at least 2 independent experiments, *p < 0.05.

As assessed by visual observation of colony diameters, growth of both MFC and AC was gradually inhibited by the addition of NaCl (Fig. 3a). When grown on medium lacking NaCl, no significant difference in colony diameter was observed between the two spore types. In NaCl-containing medium, MFC experienced drastically depressed growth, with no colonies evident at a NaCl concentration of 10% (w/v). The inhibitory effect of NaCl on colony diameter and conidiation of AC was smaller than that observed for MFC, and the differences between the two spore types were significant at 1%, 3% and 5% concentrations (*p* < 0.05) (Fig. 3b). In addition, MFC was more susceptible to NaCl, displaying an approximately 80% reduction in conidiation relative to AC, particularly at 5% NaCl (0.95 ± 0.092 × 10^6^ vs. 0.11 ± 0.083 × 10^6^ conidia ml^−1^, respectively). These results demonstrate that AC has a greater tolerance to NaCl than MFC.

### Exposure to UV radiation and cold stress

**Figure 4:**
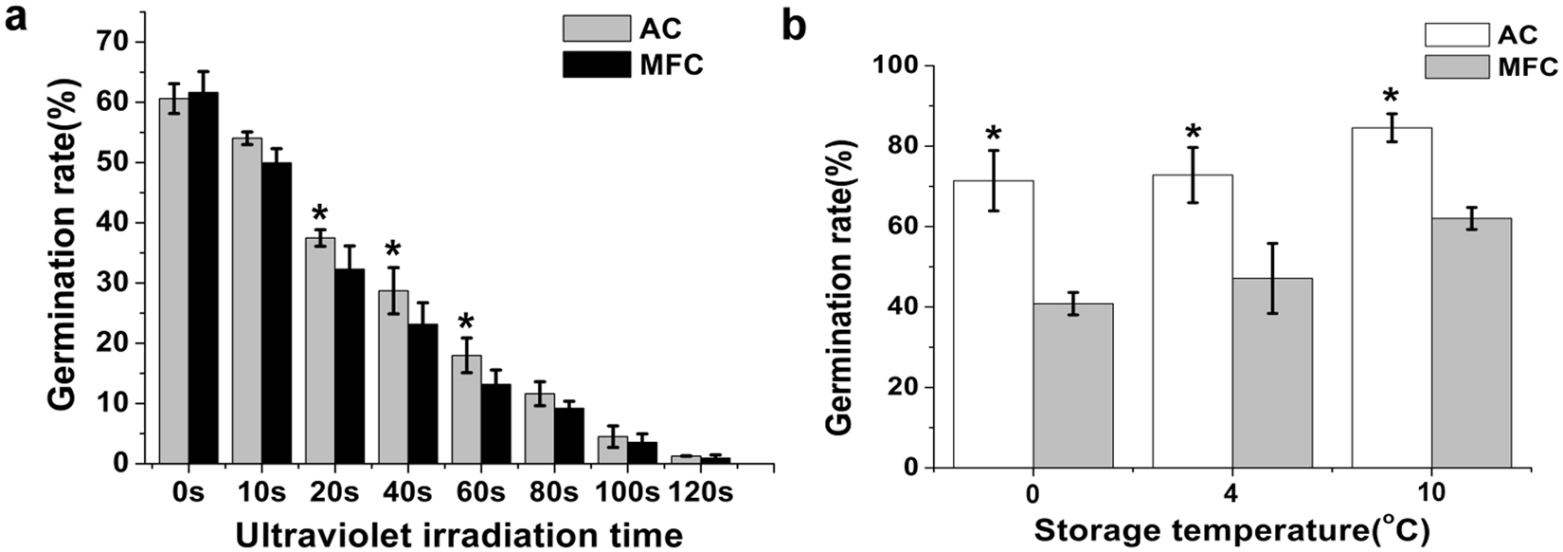
UV tolerance and cold stress test. (**a**) Bar graph quantifying conidial germination exposed AC and MFC of *Hirsutella satumaensis* to UV radiation for different duration range 0 s to 120 s. UV irradiation slowed the growth of both types of spores, but AC was less affected. Conidial germination was significantly different between AC and MFC (*p* < 0.05) following UV irradiation for 20 s (37.46 ± 1.372% vs. 32.26 ± 3.862%, respectively), 40 s (28.71 ± 3.831% vs. 23.13 ± 3.592%) or 60 s (17.97 ± 2.881% vs. 13.17 ± 2.380%). (**b**) Testing the spore viabilities of AC and MFC under cold stress (0 °C, 4 °C and 10 °C). Germination rate of MFC decrease significantly, especially at 0 °C. Statistical analysis was performed using a one-way ANOVA. Error bars are standard deviations of three trials and panels are representative of at least 2 independent experiments, *p < 0.05.

To determine the contribution of mucilage to UV tolerance, we exposed AC and MFC to UV radiation for different durations and examined the conidial germination (Fig. 4a). We found that UV irradiation slowed the growth of both types of *H. satumaensis* spores, but AC was less affected. Conidial germination was significantly different between AC and MFC (*p* < 0.05) following UV irradiation for 20 s (37.46 ± 1.372% vs. 32.26 ± 3.862%, respectively), 40 s (28.71 ± 3.831% vs. 23.13 ± 3.592%) or 60 s (17.97 ± 2.881% vs. 13.17 ± 2.380%). No conidial germination took place when either spore type was exposed to UV radiation longer than 120 s, even after incubation for 48 h. By acting as a protective film, mucilage was able to reduce direct damage to the spore surface from UV radiation and thus increase conidial germination.

The spore viabilities of AC and MFC under cold stress (0 °C, 4 °C and 10 °C) were tested (Fig. 4b). Result showed that removing of mucilage, spore germination rate decreased significantly: 0 °C (71.43 ± 7.511% vs. 40.83 ± 2.799%); 4 °C (72.80 ± 6.867% vs. 47.13 ± 8.711%); 10 °C (84.58 ± 3.476% vs. 62.03 ± 2.725%), especially at 0 °C. The lower temperature there are, the larger damage to spores. Perhaps owing to the *H. satumaensis* is a low-temperature growth species, MFC also have a higher germination rate in 10 °C. Just as a natural “antifreeze”, the mucilage with osmotic regulators making the spore cell membrane structure stabilized, protecting the protein and reducing the degree of cell damage.

### Adherence assays

**Figure 5:**
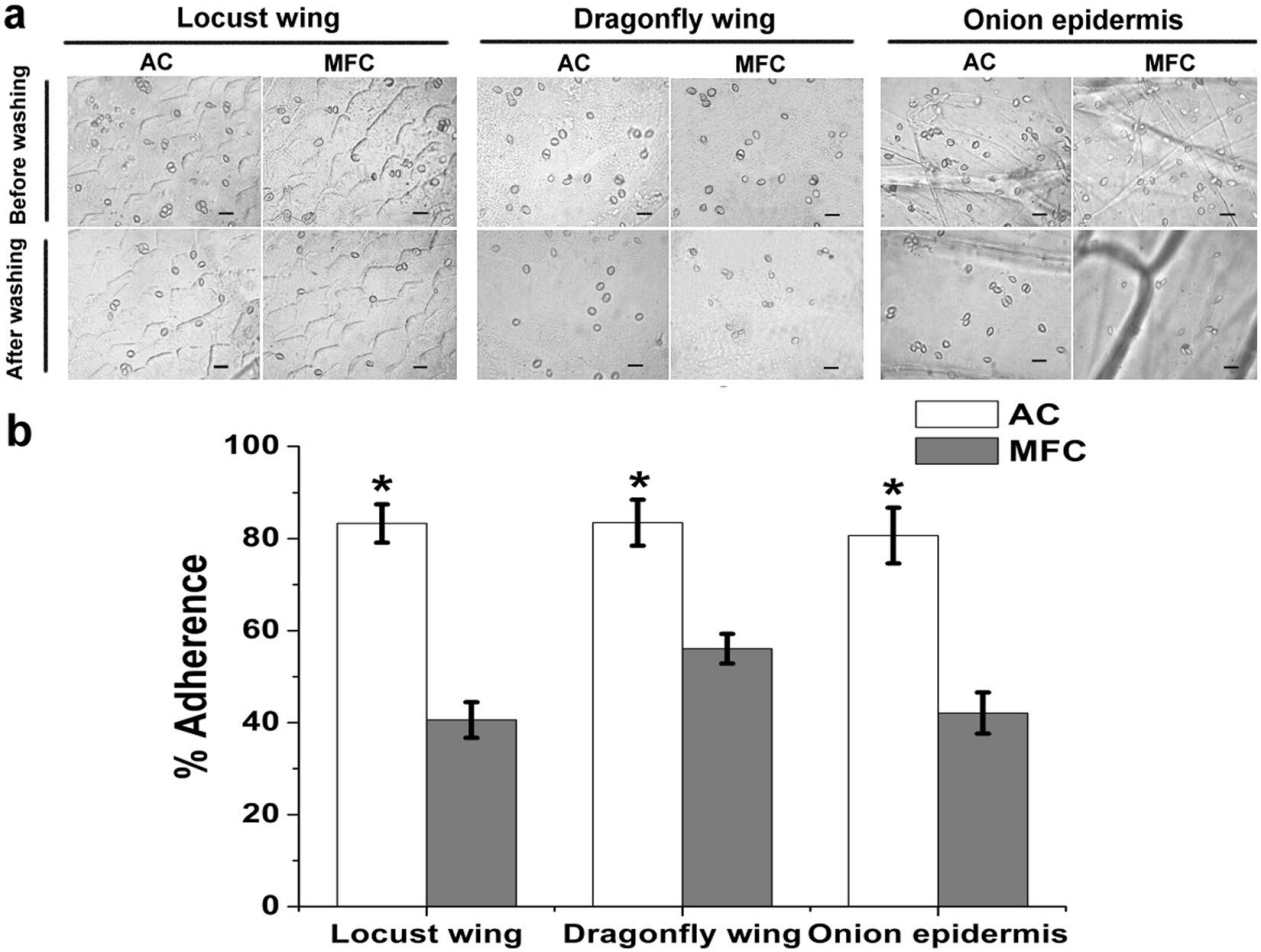
Adherence assays. (**a**) Binding of two *Hirsutella satumaensis* spores to locust wing cuticle, dragonfly wing and onion epidermis respectively in spite of washing with 0.05% Tween 20. The upper graph show the adhesion of AC and MFC to insect- and plant-derived epidermal tissue; the below display the residual spores after simulated rain erosion. Bar = 10 μm. (**b**) Bar graph quantifying adherence of two conidia to locust wing, dragonfly wing cuticle and onion epidermis. MFC display an approximately 40% reduction in adherence to the surface of three material despite washing relative to the AC. Mucilage is principally responsible for the ability of conidia to adhere to insect hosts and plant surfaces. Statistical analysis was performed using a one-way ANOVA. Error bars are standard deviations of three trials and panels are representative of at least 2 independent experiments, *p < 0.05.

To determine the contribution of mucilage to conidial adherence, we tested the adhesion of AC and MFC to insect- and plant-derived epidermal tissue (Fig. 5). Adhesion assays showed that >80% of AC adhered to locust wing cuticle (83.31 ± 4.16%), dragonfly wing cuticle (83.47 ± 5.01%) and onion epidermis (80.68 ± 6.06%) despite washing with 0.05% Tween 20. Conversely, only 40% of MFC adhered to locust wing cuticle (40.58 ± 3.88%), dragonfly wing cuticle (56.07 ± 3.23%) and onion epidermis (42.09 ± 4.47%). Mucilage is thus principally responsible for the ability of *H. satumaensis* conidia to adhere to insect hosts and plant surfaces.

### Infection bioassay

**Figure 6:**
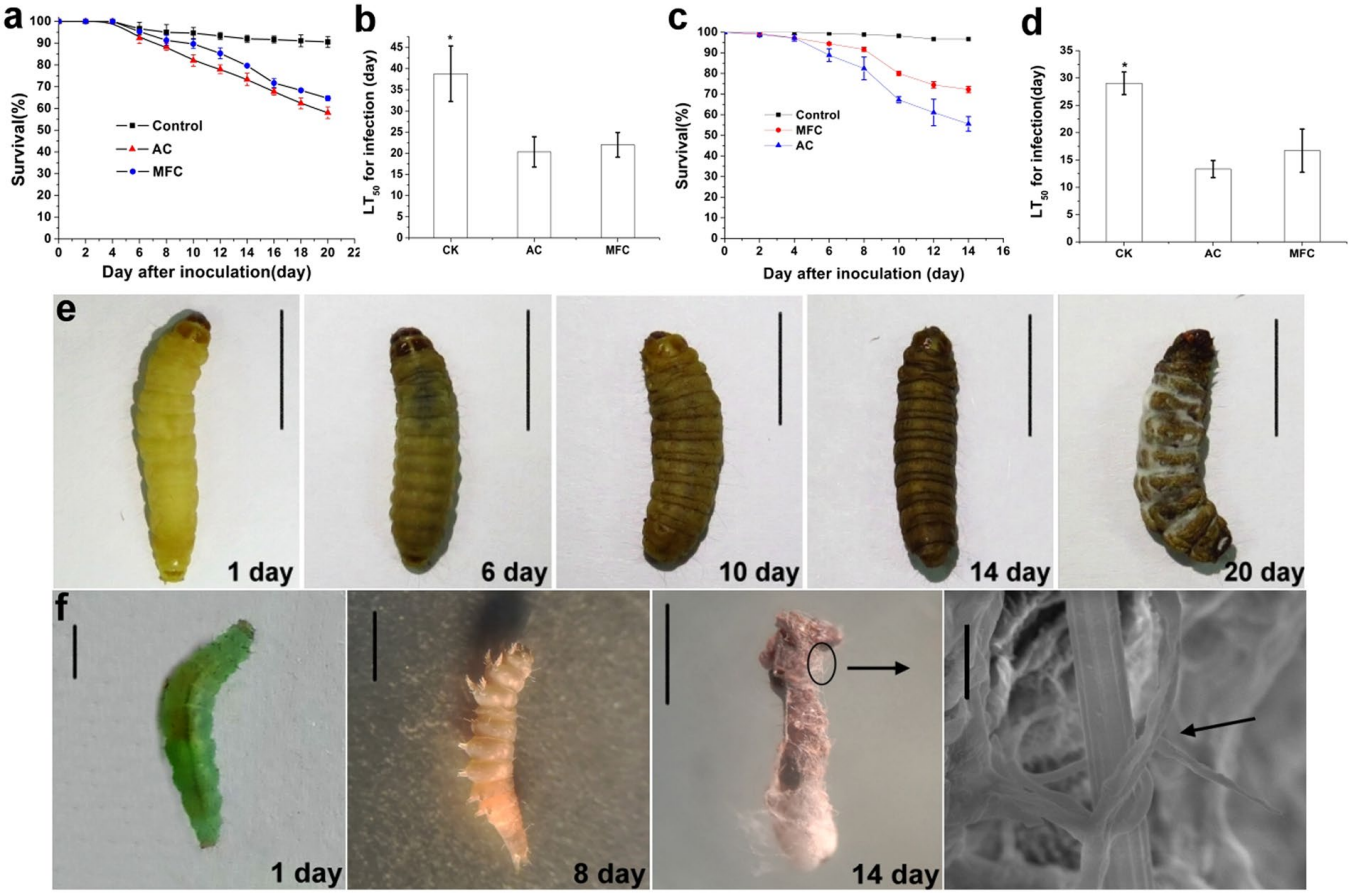
Insect bioassays. (**a**) Bar graph quantifying of survival rate of *Galleria mellonella* after application of AC and MFC of *Hirsutella satumaensis*. Relative mortality (relative to the mortality of control group, 9.43%) following application of AC (32.6% ± 1.95%) was significantly different (*p* < 0.05) than that upon infection with MFC (25.9% ± 1.50%). (**b**) LT_50_ for inoculation assay of *G. mellonella*. Compare with MFC, LT_50_ of AC is shorter though with no significant difference between them. (**c**) Bar graph quantifying of survival rate of *Pyrausta xylostella* after application of AC and MFC. *P. xylostella* was more sensitive to *H. satumaensis*, the lethality reaching 45.11% ± 3.528% (AC) and 27.45% ± 5.507% (MFC) respectively comparing with the control group (the mortality with 3.33%) after 14 days of inoculation. (**d**) LT_50_ for inoculation assay of *P. xylostella*. The LT_50_ between AC and MFC show more difference though not reaching a significant level at 0.05. (**e**) Shows cuticles change of *G. mellonella* after treatment 1 day, 6 day, 10 day, 14 day and 20 day. The cuticles of *G. mellonella* larvae change from gray to brown after infection and hyphae and conidia producing on cadaver surface at last. Bar = 1 cm. (**f**) Shows cuticles change of *P. xylostella* after treatment 1 day, 8 day and 14 day. The last one is the insect covering with hyphae and conidiogenous cell of *H. satumaensis* under SEM. The bar of insects = 2 mm; bar of SEM = 10 μm. Statistical analysis was performed using a one-way ANOVA. Error bars are standard deviations of three trials and panels are representative of at least 2 independent experiments, *p < 0.05.

The survival rate of *G. mellonella* after application of the two spore types to insect surfaces was significantly lower compared with that of the negative control group: approximately 50% survival of larvae after 20 days incubation (Fig. 6a), indicating low virulence towards the insects, as well as the statistical result of LT_50_ for infection (Fig. 6b). Nevertheless, the host fatality rate between AC and MFC were different. Relative mortality following application of AC (32.6% ± 1.95%) was significantly different (*p* < 0.05) than that upon infection with MFC (25.9% ± 1.50%). But the LT_50_ have an opposite result. In both bioassays, cuticles of *G. mellonella* larvae changed from gray to brown after infection, and hyphae and conidia emerged from insect cadavers approximately 20 days after infection with either AC or MFC (Fig. 6e). *P. xylostella* was more sensitive to *H. satumaensis*, the lethality reaching 45.11% ± 3.528% (AC) and 27.45% ± 5.507% (MFC) respectively comparing with the control group after 14 days of inoculation (Fig. 6c). The LT_50_ between AC and MFC shows more difference though not reaching a significant level at 0.05 (Fig. 6d). Similar with *G. mellonella*, the cuticles of *P. xylostella* larvae changed from cyan to brown after infection. According to those differences in lethality between AC and MFC on two kinds of insect determine that the mucilage has a positive effect on fungal virulence (Fig. 6f).

## Discussion

In this study, we comprehensively investigated the morphology and ecological function and significance of mucilage from the entomopathogenic fungus *H. satumaensis*. According to our results, mucilage chiefly contributes to AC surface hydrophobicity but not colony morphology, improves conidial viability under unfavorable growth conditions, including extreme temperature, high osmotic pressure, UV radiation and cold stress, and enhances adhesion and host pathogenicity. These findings also suggest that mucilage contains substances such as oligosaccharides and proteins, consistent with previous reports that high-molecular-weight components of proteins, invertase, esterase and polysaccharides are one of the main constituents of mucilage^18, 26–29^.

The *Hirsutella* genus are very special asexually-reproducing pathogens of insects within *Ophiocordycipitaceae*, which distinguished from other entomopathogenic fungi such as *Beauveria* and *Metarhizium* by their slower growth, lower growth temperature, reduced sporulation, host specificity and spores covered by a thick mucilage layer^30^. Differences in physiological structure may lead to the special on mechanism of infection or environmental adaptability. One interesting question is understanding how the latter characteristic has affected the ability of *Hirsutella* species to reproduce and persist. From an evolutionary point of view, *Ophiocordyceps*, a monophyletic group, emerged earlier (109–138 Millions of years ago, Mya) than Cordycipitaceae (91–146 Mya) and Clavicipitaceae (95–144 Mya), with *H. minnesotensis* diverging from *O. sinensis* around 23.9–33.9 Mya^31–33^. This sequence of events indicates that spores containing more mucilage appeared early during the evolutionary period. To adapt to long-term survival in a frigid environment, *Hirsutella* evolved a unique adaptive covering, mucilage, which functions as an appendage and protector of spores with important ecological significance for species dispersal and evolution. Our recent findings showed that some species of *Hirsutella* distributed in two or more different habitat niche, where temperature difference could reach to 20 °C or even the cold areas under 0 °C. It is surprising that the strong ecological adaptability and also confirms the positive effect of mucilage for spores from another side. In fungi with a narrow host range, a quality-based host-infection strategy can be more effective than a quantitative one. According to previous study, the infection rate of *H. rhossiliensis* onto nematodes can be increased to more than 90%, a significant improvement, using the single-spore inoculation technique^34^. This finding demonstrates that a single spore of *Hirsutella* with a high activity, which distinct from the large sporulation of broad-host-range pathogens (such as *Beauveria*), whereas many of them lack the ability to infect hosts without nuclei. To summarize, *Hirsutella* species use a “K-strategy” for propagation, while the mucilage of their spores is a product of adaptive evolution.

Numerous studies on mucilage of plants or some plant pathogenic fungi have suggested that mucilage frequently serves to protect spore vitality or acts as an antidesiccant^15, 17, 35^. Some fungi produce an extracellular matrix that provides some level of UV protection to the spore^6^. Mucilage has also been suggested to protect the mycelium against the diffusion of Cu^2+^ ions and fungicide toxicity through the presence of proline-rich proteins that have a high binding affinity for a variety of phenols, enabling continued growth or survival of the fungus^4, 36^. Our study yielded similar results. At 15 and 30 °C, the growth of AC was greater than that of MFC, with conidiation between the two spore types significantly different. At optimum temperatures ranging from 20 to 25 °C, in contrast, MFC colony diameters were significantly larger than those of AC; a NaCl tolerance test showed a similar trend. Furthermore, the viability of AC following UV irradiation and sustained low temperature stress was generally higher than that of MFC. It is unclear why non-mucilaginous spores have a higher germination rate under suitable conditions. Under optimum conditions, such spores rapidly and directly contact and identify the medium, thereby perhaps promoting better growth; under unfavorable conditions, however, such rapid germination would be disastrous. Various authors have detected germination inhibitors in fresh mucilage^37–39^. The spore matrix has been found to have dual roles, namely, inhibition of premature germination and protection of spores during adverse conditions^6^.

The attachment of infective fungal propagules is essential for the initiation of mycosis^40^. In this regard, surface properties including hydrophobicity of the fungal cells form the basis for the host–pathogen interaction. A study of the attachment properties of *B. bassiana* cell types revealed that AC with more hydrophobicity adhere rapidly to hydrophobic surfaces, but poorly to weakly to polar surfaces. Even when spores rapidly attach to hydrophilic surfaces, the effect cannot be consolidated and the spores can be rapidly washed off^5, 41^. Cell surface hydrophobicity is associated with increased virulence of *Candida* strains, and the hydrophobic rodlet layer of *Aspergillus* conidia appears to confer protection against host immune reactions^42–44^. In the present study, we first demonstrate that the mucilage was mainly contributed to surface hydrophobicity and enhanced attachment to various substrates of *H. satumaensis*. Removal of the mucilage by elution caused an approximately 40% reduction in adherence to insect- and plant-derived epidermal tissues, which suggests that *H. satumaensis* conidial mucilage possesses many molecules that adhere to plant and insect surfaces. As mucilage contributes to adherence and is associated with *H. satumaensis* pathogenicity, it is unsurprising that its removal slightly reduced virulence of the conidia.

As part of the current focus on the reduction of chemicals in agriculture in response to increased insect resistance to pesticides, entomopathogenic fungi have been investigated as promising alternative insect-pest biocontrol agents^45–47^. For it medicinal and edible properties, *Hirsutella* can be developed as a safe, host specific, eco-friendly control factor for arthropod. Therefore, mucilage is an ideal biological material that can be used to improve the stability of pesticides exposed to UV radiation or other environment stresses. In simple terms, mucilage on spores is comparable to a film coating on plant seeds. Furthermore, formulations that mimic mucilage attachment and adherence can be developed and used to enhance infection rate and host pathogenicity. The most attractive future application of mucilage is as a plant moisturizer to extend the shelf life under low temperature and virulence of commercially available pathogenic fungi when applied in biocontrol. Moreover, mucilage may also be used as a type of biological glue for medical treatments and pharmaceutical applications. Further study in this direction should reveal the components expressed genes of mucilage that are involved in fungal spore–insect surface mechanisms and the molecular and regulatory mechanism responsible for the production of mucilage and stress resistance.

In summary, studying of spore mucilage has an important significance for understanding of the mechanism of interaction between pathogenic fungi and host, important *Cordyceps* species artificial cultivation and biological control. Our results demonstrate that conidial mucilage plays positive roles in the ecological adaptability and pathogenicity of *H. satumaensis*. This study has provided new insights that may be useful for future applications of mucilage as an antidesiccant or reinforcing agent in biopesticide formulations that exploit entomopathogenic fungi.

## Material and Methods

### Fungal strain

*H. satumaensis* strain GZUIFR**-**Hir201012JC (host: *Bombyx mori* Linaeus) was obtained from the Institute of Fungi Resources, Guizhou University, fungal cultures were maintained on potato dextrose agar (PDA).

### Prepare for two conidia and colony culture

The strain was routinely grown on PDA. Plates were incubated at 22 °C for 14 days and aerial conidia were harvested by flooding the plate with sterile 50 mM pH 7.2 Phosphate buffer. Conidial suspensions were filtered through four layers of lens paper and final spore concentrations were resuspended to the desired concentration (typically 10^6^–10^7^ conidia ml^−1^) using a haemocytometer. The half of harvested aerial conidia were resuspended to concentration of 10^8^ cells ml^−1^ using 50 ml sterile 50 mM pH 7.2 Phosphate buffer with glass beads, then added 1 ml concentration of 0.1% *β*-mercaptoethanol as reducing agent, and whirligig 12 h at 120 rpm/min, 4 °C. The mucilage-free conidia (MFC) were harvested by centrifugation (10 000 g, 10 min, 4 °C), then washed two times with sterile dH_2_O, and were resuspended to concentration of 10^7^ conidia ml^−1^ ^48–51^.

### Determination of conidia surface hydrophobicity

Microbial adhesion to hydrocarbons (MATH) assay. Conidia surface hydrophobicity was determined essentially as previous described^25, 52^. Briefly, AC and MFC were washed into PUM buffer (per litre: 22.2 g K_2_HPO_4_, 7.26 g KH_2_PO_4_, 1.8 g urea, 0.2 g MgSO_4_. 7H_2_O, final pH 7.1). Fungal cell suspensions were adjusted to OD_470_ 0.4 and dispensed 3 ml into acid-washed glass tubes (10 ml). Hexadecane (300 μl) was then added to each tube and vortexed three times for 30 s respectively. The vortexed tubes were allowed to stand at room temperature for 15 min before the hexadecane phase was carefully removed and discarded. Tubes were then cooled to 4 °C and any residual solidified hexadecane was removed. The tubes were then returned to room temperature and the A_470_ of the resultant cell suspensions was determined. The hydrophobic index was calculated using the following equation: (A_470, contro_ − A_470, hexadecane treated_)/(A_470, control_).

### LM and SEM observations

For light microscope (LM) observations and imaging, two spores were stained with lactic acid phenol cotton blue solution and observed with optical microscope (OM, BK5000, OPTEC, USA) respectively.

Scanning electron microscope (SEM) observation was determined according to Cao and colleagues^53^. Briefly, the collected samples were fixed with 4% glutaraldehyde at 4 °C overnight, then washed three times with phosphate buffer solution (PBS) (137 mM NaCl, 2.7 mM KCl, 8.1 mM Na_2_HPO_4_, 1.5 mM KH_2_PO_4_, pH 7.4) three times, 10 min/times; Fixed conidia were dehydrated using 50%, 70%, 90% and 100% acetonitrile, 10 min/each level; dehydrated with 100% acetonitrile twice at last. Placed the samples to sprayed gold, conidia and mucilage were examined with SEM (S–3400N, HITACHI, Japan) and photograph.

### Non-biotic stress tolerance assay of conidia mucilage

To analyse mucilage effect of vegetative growth on medium with cell stress chemicals, the AC and MFC were grown on PDA agar plate, where supplemented with cell stress chemical NaCl (1%, 3%, 5%, 10%, 20%, w/v) respectively. Aliquots of 5 μl conidial suspension (1 × 10^7^ conidia ml^−1^) were pipetted onto plates, which were incubated at 22 °C and inspected regularly for 14 days for colony diameter and conidiation per 1.33 cm^2^.

For heat tolerance experiment, aliquots of 5 μl two spores conidial suspension (1 × 10^7^ conidia ml^−1^) were pipetted onto plates, then were incubated at 15 °C, 20 °C, 25 °C, 30 °C, 35 °C respectively and inspected regularly for 14 days for colony diameter and conidiation per 1.33 cm^2^.

For ultraviolet radiation tolerance, two spores were aliquoted onto PDA medium surface covered with cellophane as described above. After inoculation, the spores were exposed at UV radiation for 10 s, 20 s, 40 s, 60 s, 80 s, 100 s, 120 s respectively. The plates were then incubated for 2 days in the dark immediately at 22 °C before observing and count germination using a microscope^54^. All experiments were carried out in triplicates.

In order to investigate the spore viabilities of AC and MFC under cold stress, the test was implemented according to Mondal and Parbery^6^. Two spores were transferred to sterile No. 1 Whatman filter papers and dried for 15–20 min until there was no visible excess water. In each case, the filter papers and spores were placed in loose-capped McCartney bottles and stored at 0 °C, 4 °C and 10 °C for the 7 days respectively. After rehydration of dried spores, an aliquot of 200 μL of spore suspension was transferred to cellophane squares placed on PDA, incubation for 48 h and counting the proportions of germinated spores in each treatment. The germination status of at least 200 spores per treatment was recorded from randomly selected fields under a binocular microscope at 100× magnification. Each experiment was repeated in triplicates.

### Adherence assays

Method referenced Wang *et al*.^21^ described and slight changed. Briefly, suspensions of AC and MFC were prepared in 0.05% Tween 20 at a concentration of 1 × 10^7^ conidia ml^−1^, washed twice with sterile water, and resuspended in sterile water for use. Locust hind wings, dragonfly wings, and epidermis peeled from pieces of onion (1–0.5 cm) were sterilized in 37% H_2_O_2_ for 5 min, washed twice in sterile water, immersed in spore suspensions for 20 s, and placed on 0.7% water agar. After incubation for 8 h (to induce spore swelling and initiation of germination), the number of conidia in five objective fields was counted under a light microscope before and after washing out the less adherent conidia in 0.05% Tween 20 for 30 s. Adherence by two conidia on the different materials was estimated using the average number of conidia per objective field after washing compared to the average count before washing (always >300 conidia per treatment). Experiments were conducted in triplicate, and each experiment was repeated at least twice.

### Insect bioassays

Fourth instar nymphs of *Galleria mellonella* Linnaeus (Lepidoptera, Pyralidae) and third instar of *Plutella xylostella* Linnaeus (Lepidoptera, Plutellidae) were used for bioassays. Assays were performed using sterile conical flasks with 10 g silicon (IV) oxide. Each treatment group had 30 insects in triple replicates, and experiments were performed twice independently. The AC and MFC were assayed at 2.0 × 10^7^ conidia ml^−1^, and aliquots of 5 ml conidial suspension were pipetted into flasks respectively, mixing with sterile silicon oxide, using degrease cotton moisturizing, then putting insects to flask, incubated at 22 °C and inspected regularly for the number of dead larvae^55^. Dead insects were moved to containers with a wet paper towel and incubated at 22 °C to determine emergence of hyphae and conidia from insect cadaver. Survival rate and mean median lethal time (LT_50_) was estimated and compared among the AC, MFC and the negative control group. A negative control group submerged heat killed conidia was used, briefly, the two mixed spores were denatured by wrapping the conidia in aluminium foil and autoclaving for 15 min at 121 °C^56^.

### Data analysis

Colony diameter, conidiation, germination rate, adherence index, survival rate and relative mortality in bioassay were statistically analysed using an analysis of variance one-way model (Oringin Pro 8.0; OringinLab, Massachusetts, USA). LT_50_ were analyzed using an ANOVA one-way model with SPSS 21.0 program (SPSS Inc, Chicago, IL, USA). Tukey’s honestly significant difference test was used to separate means at 0.05. Data were represented as mean ± SD.

## Electronic supplementary material

The conidial mucilage, natural film coatings, is involved in environmental adaptability and pathogenicity of Hirsutella satumaensis Aoki
