# Supplementary material for: The conidial mucilage, natural film coatings, is involved in environmental adaptability and pathogenicity of Hirsutella satumaensis Aoki
Source: Sci Rep. 2017 May 2;7:1301. doi: 10.1038/s41598-017-01368-1 (PMC5431061; doi:10.1038/s41598-017-01368-1)
Supplement: Supplementary file 1 — The conidial mucilage, natural film coatings, is involved in environmental adaptability and pathogenicity of Hirsutella satumaensis Aoki [file 41598_2017_1368_MOESM1_ESM.pdf]

The conidial mucilage, natural film coatings, is involved in environmental adaptability and pathogenicity of *Hirsutella satumaensis* Aoki

Jiaojiao Qu[1, 2], Xiao Zou[2*], Jianping Yu[3], Yeming Zhou[4]

[1] Key Laboratory of Green Pesticide and Agricultural Bioengineering, Ministry of Education, Guizhou University, Guiyang, 550025, China

[2] Institute of Fungus Resources, Guizhou University, Guiyang, 550025, China

[3] College of Life Sciences, Guizhou University, Guiyang, 550025, China

[4] Institute of Entomology, Guizhou University, Guiyang, 550025, China

[*] Corresponding author. E-mail address: xzou@gzu.edu.cn

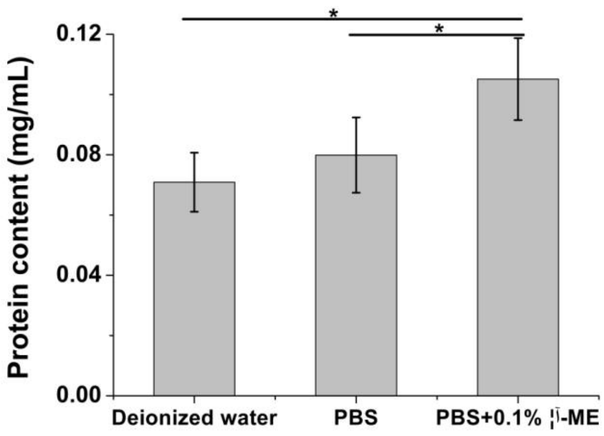

Supplementary Fig.1 Protein content of mucilage from *Hirsutella satumaensis* extraction with three solvents. After adding 0.1% β-mercaptoethanol as a reducing agent, the protein content of mucilage extracted from spores with PBS buffer was highest comparing with deionized water and PBS. It indicating that addition of reducing agent could help the separation of protein from the mucilage. Error bars are standard deviations of three trials. * means $p<0.05$.

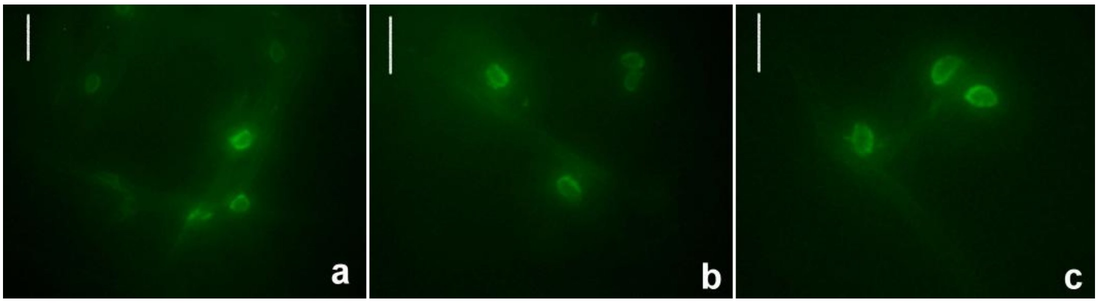

Supplementary Fig.2 Fluorescence images of the Alexa Fluor 488-labelled lectins concanavalin A binding profiles of *Hirsutella satumaensis* aerial conidia. From the picture, the bright green fluorescence in mucilage around the spores could be observed clearly, which is indicate that the mucilage containing substances such as α-mannopyranosyl and α-glucopyranosyl residues at least. Bars=10μm.
